# Refugia Persistence of Qinghai-Tibetan Plateau by the Cold-Tolerant Bird *Tetraogallus tibetanus* (Galliformes: Phasianidae)

**DOI:** 10.1371/journal.pone.0121118

**Published:** 2015-03-30

**Authors:** Bei An, Lixun Zhang, Naifa Liu, Ying Wang

**Affiliations:** 1 School of Basic Medicine Sciences, Lanzhou University, Lanzhou, China; 2 School of Life Sciences, Lanzhou University, Lanzhou, China; 3 Gansu Key Laboratory of Biomonitoring and Bioremediation for Environmental Pollution, Lanzhou, China; 4 Pediatric Research Institute, Qilu Children's Hospital of Shandong University, Ji'nan, Shandong, China; Sichuan University, CHINA

## Abstract

Most of the temperate species are expected to have moved to lower altitudes during the glacial periods of the Quaternary. Here we tested this hypothesis in a cold-tolerant avian species Tibetan snowcock (*Tetraogallus tibetanus*) using two segments of mitochondrial gene (a 705bp Cytochrome-b; abbrev. *Cyt-b* and an 854 bp Control Region; abbrev. CR) and eight microsatellite loci by characterizing population differentiation and gene flow across its range. Combined (*Cyt-b* + CR) datasets detected several partially lineages with poor support. Microsatellite data, however, identified two distinct lineages congruent with the geographically separated western and central regions of Qinghai-Tibetan Plateau (QTP). The phylogeographic patterns that we observed might be explained by a combination of vicariance events that led to local isolation of *T*. *tibetanus* during warm periods and range expansions and population intermixing during cold periods. The results of this study add to our knowledge of population differentiation and connectivity in high altitude mountain ecosystems.

## Introduction

Historical refugia have long been thought vital in shaping contemporary patterns of biological diversity [1]. The previous phylogeographic and palaeontological studies on the biota of Qinghai-Tibetan Plateau (QTP) and adjacent regions have revealed a complicated scenario of glacial survival in refugia [2–6]. Further, there is ample evidence to suggest that many species responded individually to climate changes as such single refugium is unlikely to be suitable for all of them [3–5,7]. Shi[8] also suggested some cold-tolerant animals or herbs could have persisted in ice-free areas of the central plateau region during glacial maximum. However, there is a lack of detailed phylogeographic studies on cold-tolerant species from the Tibetan Plateau [4,5,9,10]. Clearly, further phylogeographic studies of QTP alpine species are required to obtain a better understanding of their recent demographic history and, in particular, whether these species survived in refugia on the central of QTP during Pleistocene glaciations.

Cold-tolerant avian species with limited dispersal capabilities present potentially good candidates to test whether they have persisted in central refugia or retreated to the edge of QTP during Pleistocene glaciations. The Tibetan snowcock *Tetraogallus tibetanus* [11] is the only species distributed at the high altitudes in Galliformes group [12]. Tibetan snowcock, evaluated as Least Concern (IUCN/SSC www.redlist.org 2013) and as class II Protected Status under Chinese law, feeds mainly on the roots, shoots, grass, leaves and insects, and inhabits fluvial rocky hills, alpine meadows, hilly pastures, and barren shrubby grasslands[13]. When the Tibetan snowcock’s current range in China is considered, it currently distributes disjunctly in the western, central and eastern of the QTP, which is either glaciated or laid close to the limits of the ice sheets. Tibetan snowcock is relatively sedentary and has restricted to low-temperature [13]. In comparison to the refugia of other species in the glaciations, distribution of Tibetan snowcock might have fluctuated repeatedly during Pleistocene glaciations and interglaciations. Given that relatively few studies have been made on fauna and flora from the western plateau edge of QTP [14], this study aims to improve the phylogeographic knowledge with reference to this part of the country. The following questions were investigated:(1) did Tibetan snowcock retreat to the plateau edge and then recolonise the interior of the plateau, like other species, or did the current populations persist in the interior of the plateau *in situ*? (2) Have recent climate-induced habitat shifts shaped patterns of population connectivity and gene flow in this evolutionary hotspot [15–18]?

## Materials and Methods

### Collection of Birds

**Fig 1:**
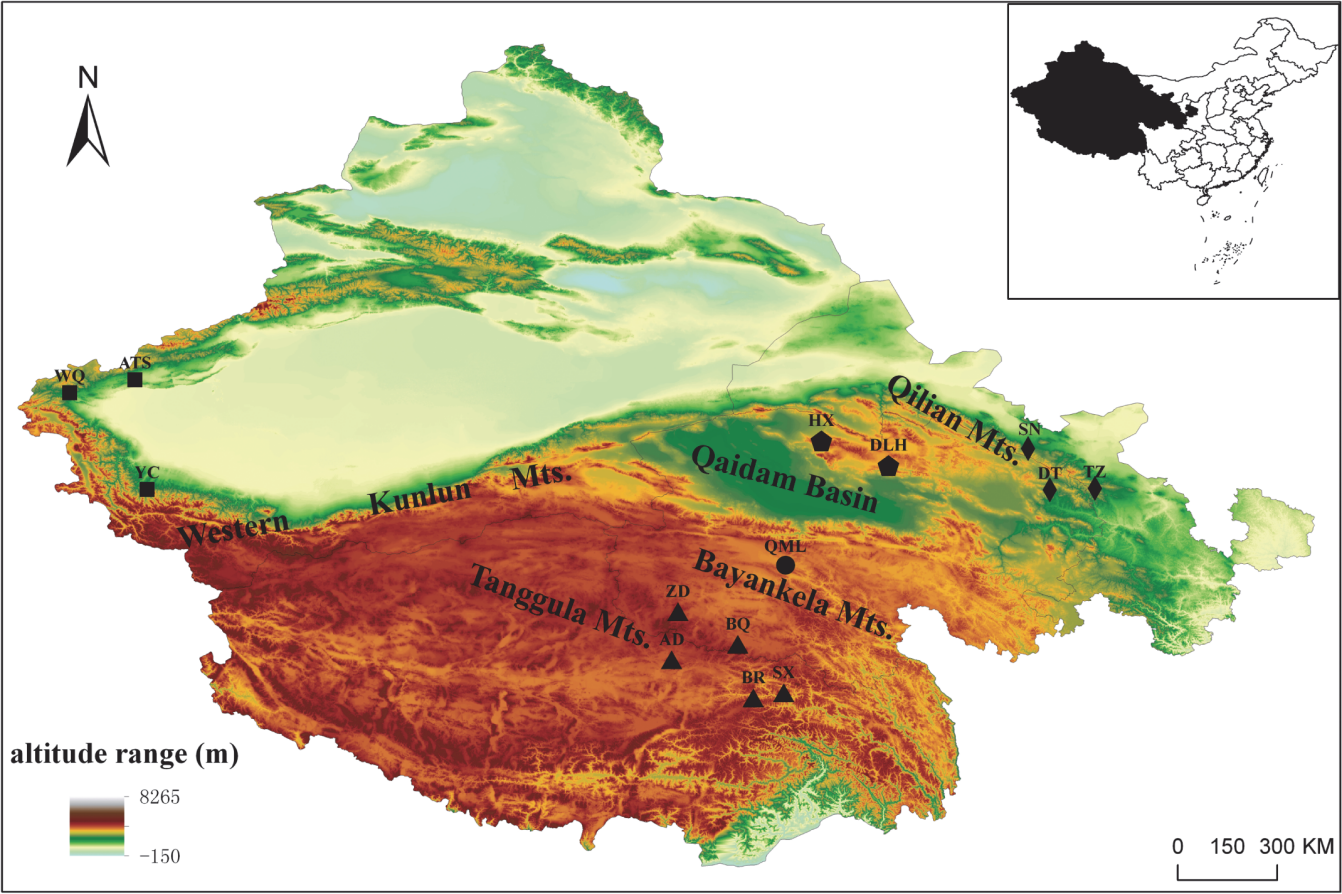
Locations of the 14 sampled populations of the Tibetan snowcock *Tetraogallus*. *tibetanus* in Qinghai-Tibetan Plateau of western China. Sampling sites: WKL group: Atushi (ATS), Yecheng (YC) and Wuqia (WQ); QLS group: Tianzhu (TZ), Sunan (SN) and Datong (DT); QDM group: Haixi (HX) and Delingha (DLH); BKL group: Qumalai (QML); TGL group: Zhiduo (ZD), Biru (BR), Anduo (AD), Baqing (BQ) and Suoxian (SX).

The study of high altitude species requires hard and difficult fieldwork. In total, 80 individuals of Tibetan snowcock were collected from 14 sampling sites in the QTP at elevations from 3700 to 6000 m a.s.l. These sites cover most of the distribution range of the species. The sample size of each population is shown in S1 Table. According to their geographical origin, the samples were divided into five regional groups: QLS (Qilian Mountain region), QDM (Qiadam Basin region), BKL (Baryan Har Mountains region), TGL (Tanggula Mountains region) and WKL (west Kunlun Mountains region) (Fig 1 and S1 Table). The research was conducted under permission, to collect snowcock and to work in several protected areas, by the Forest Department of Gansu(China, Gansu), Xinjiang(China, Xingjiang Autonous Region), Xizang (China, Xizang Autonomous Region). This study was approved by the Animal Ethics Committee of the Chinese Academy of Lanzhou University (permit number: SCXK Gan 2013–0002). All avian specimens were collected by cage-trap method and mist net and handled in accordance with good animal practice as required by the Animal Ethics Procedures and Guidelines of the People's Republic of China. Muscle and liver samples, which are taken after euthanasia by inhaled anesthetic through Carbon dioxide, were immediately stored in 95% ethanol at -18°C. Voucher specimens are held in the Lanzhou university nature museum.

### Molecular Analysis

#### DNA extraction, sequencing and genetyping

Total genomic DNA was extracted from the samples using the DNA whole genome kit Qiagen, Inc., Valencia, CA). Two mitochondrial DNA (mtDNA) fragments from the Cytochrome-b (*Cyt-b*) and Control Region (CR) were PCR-amplified. Two new pairs of primers were designed for Tibetan snowcock using Primer3 [19]. The list of primer pairs, corresponding annealing temperatures and product sizes are given in S2 Table. Each round of PCR reactions also included one negative control to check for contamination. PCR products were visualized on a 1.5% agarose gel, purified and directly sequenced in both directions on 3730xl DNA. Final alignments were constructed by eyes using DNASTAR software (Version 7.10, DNASTAR, Madison, USA) employing 80 individuals. All samples of WKL group were genotyped at eight polymorphic microsatellites primers that were isolated originally from the chicken (*Gallus gallus*) genome: (MCW135, MCW323, AB121114, GUJ0029, UBC0002, UBC0005, UBC0006 and GUQ0001). Primer sequences and information on markers can be retrieved from http://www.ncbi.nlm.nih.gov/sites/entrez. The published microsatellite data from the other 4 groups (QLS, QDM, BKL and TGL group) have been involved in this study[20]. Fragment lengths were analyzed with the internal size marker GENESCAN-500 ROX (Applied Biosystems), and scored with GENEMARKER 3.7 (SoftGenetics).

### Genetic diversity and phylogeographic structure

For mitochondrial data, we identified haplotypes and calculated standard genetic indices (for example, haplotype diversity (h) and nucleotide diversity (π) for each population and all populations combined diversity using DNASP 5.0 [21].

For microsatellite data, we estimated the standard genetic diversity for each site using the following indices: the number of alleles at each locus (NA), average allelic richness (A_R_), observed heterozygosity (H_O_), heterozygosity expected from the Hardy-Weinberg assumption (H_E_) and exact tests of linkage disequilibrium (LD) between pairs of loci for each population were calculated with ARLEQUIN 3.5 [22] and FSTAT 2.9.4[23].

A hierarchical analysis of molecular variance (AMOVA) was performed to compare levels of mitochondria and microsatellite data genetic diversity within and among several possible population groupings of Tibetan snowcock using ARLEQUIN. Genetic differentiation between regional groups was evaluated based on Pairwise values of F_ST_. The statistical significance of the estimates was assessed after 10,000 permutations. Gene flow (Nm) was calculated to ascertain the conditions of gene communication among populations, and was estimated as follows: Nm = (1- F_ST_)/4 F_ST_[24]_._ F_ST_ and Nm were calculated using the software ARLEQUIN.

Evolutionary relationships among all haplotypes were performed by constructing phylogenetic trees using Bayesian (BA) Markov chain Monte Carlo (MCMC) phylogenetic analyses [25]. The computer program jMODELTEST 2.1.1[26] was used to select the best-fit model of evolution. For Bayesian analyses, four independent MCMCs were initiated with random starting trees and each run for 5×10^6^ generations, sampling every 100 generations. A 50% strict consensus tree was computed for the 1000 bootstrap trees.

Phylogenetic analyses were also run in PAUP 4.0b10 [27] using maximum Likehood (ML) for Cyt-b and D-loop data. ML analyses were conducted using heuristic searches with tree-bisection-reconnection branch swapping, with 100 random input orders of taxa and a maximum of 1000 trees retained during each search. Indels were treated as a fifth state in ML analyses. For ML, bootstrap analyses of 1000 replicates were performed using the same settings.

The phylogenetic trees were rooted using homologous sequences derived from the Himalayan snowcock (*Tetraogallus himalayensis)* (GenBank accession GQ343530.1; GQ343542.1). We further constructed unrooted haplotype networks using the median-joining algorithm [28] as implemented in Network v4.6.1.1 (http://www.fluxus-engineering.com). This method allows the visualization of mtDNA haplotype relationships and frequencies.

We used two methods to identify genetically distinct groups among microsatellite genotypes. First, a Factorial Correspondence Analysis (FCA) [29] was employed to cluster individual microsatellite profiles in a multidimensional space using the algorithm implemented in GENETIX 4.05 [30]. We further tested for genetic structure (assuming no prior imposing spatial information for the snowcock samples) using a Bayesian clustering method, which was implemented in STRUCTURE v2.3 [31,32]. An admixture model with correlated allele frequencies was used and performed 300,000 MCMC steps with 200,000 burn-in steps. We conducted 10 independent runs for each K-value (K = 1–14) for the entire dataset, the average proportion of membership (qi) of the sampled populations was assessed in each detected cluster. Then each individual was assigned to the detected clusters using a threshold qi > 0.80, according to the results from the simulation procedures [33] for the assignment of individuals genomes to one cluster or in the case of admixed individuals, jointly to two or more clusters if the proportion of membership to each one was qi < 0.80.

### Historical demography

The dynamics of population size fluctuations were estimated using the Bayesian skyline plot (BSP) method implemented in BEAST. This approach incorporates uncertainty in the genealogy by using MCMC integration under a coalescent model, in which the timing of dates provides information about effective population sizes through time. Chains were run for 100 million generations and the first 10% discarded as ‘burn-in’. The substitution model was selected according the result of jMODELTEST. We applied 10 grouped coalescent intervals and constant growth for the skyline model. Because no fossil data were available to calibrate the mutation rate, we assumed a conventional molecular clock for the avian mtDNA *Cyt-b* gene (1.6 × 10^–8^ per site per year) [34]. Demographic history through time was reconstructed using Tracer 1.5 [35].

Demographic histories of each clade were inferred by pairwise mismatch distribution analyses [24] and computed under a population growth-decline model in DNASP version 5.0. We also tested the hypothesis of population expansion calculating Fu’s neutrality statistic *Fs* [36] and Tajima's D [37] test in DNASP version 5.0 and mismatch distributions. Population expansions can cause significant negative departure of Tajima’s D from zero. And recent demographic expansions lead to a signicantly negatie *Fs* value.

### Historical Biography

To determine whether populations remained isolated in individual habitats (multiple refugia), or merged into a single gene pool (single refugium) during glaciations and interglaciations, we used coalescent simulations in MESQUITE 2.75[38] to test two biogeographic hypotheses. First hypothesis assumed that five groups (QLS, TGL, QDM, BKL and WKL) diverged since late Pleistocene, after expanded from single refugium. Second hypothesis assumed that five populations stayed in a single refugium during Pleistocene, subsequently diverged to two lineages (WKL group versus other four groups), which corresponds the genetic structure detected by microsatellite data. We used Paup* 4.0b10 [27] to reconstruct trees from the simulated gene matrices, and the S-values for these trees (the minimum number of sorting events required to explain the population subdivision) were recorded. For all coalescent simulations, absolute time (years) was converted to coalescent time (generations) assuming a generation time of 2 year for Tibetan snowcock[20]. We tested whether the observed genealogies were consistent with the given models by comparing the S-value of the empirical ML genealogy with those of the simulated genealogies.

For coalescent simulations, the effective population size (*Ne*) using the θ-values calculated by the ML and coalescent-theory approach in the program MIGRATE 1.7.3[39,40]. This approach assumes the Wright–Fisher model, and in order to find the best-fit model with which to obtain convergent and consistent results, we ran the program for several replicates and used different parameter combinations. The results of analyses were accepted if the 95% confidence intervals (CIs) of one run included the estimated ML value of the other runs and results of multiple runs were similar. Finally, the following parameters were used for our analyses: 10 short chains of 1,500,000 steps followed by three long chains of 15,000,000 steps; chains were sampled every 100 steps following a burn-in of 100,000 steps, and default settings were used for the initial estimate of the θ-value. We converted the θ-value to *Ne* using the formula θ = 2 *Neμ* using same mutation rate in demography time estimate. When running the coalescent simulations, we set the overall *Ne* to equal the empirically estimated values, and constrained the Ne of the putative refugial population to a size proportional to the overall empirically estimated *Ne*.

## Results

### Genetic Diversity and Phylogeographic Structure

All sequences are accessible at GenBank (Accession nos: JX136799- JX1368336 for CR and JX136834—JX136846 for *Cyt-b*). A total of 1589 bp of mtDNA was obtained, which contained 136 polymorphic sites, 124 of which were parsimony informative. These polymorphic sites defined 49 unique haplotypes, 36 of which occurred only once. 7 haplotypes were shared among individuals within the same population and six haplotypes were shared between populations. Within sampling locations, haplotype diversity values were from 0.73 to 0.98, and nucleotide diversity ranged from 0.0019 to 0.036 (Table 1).

**Table 1:** Estimates of genetic diversity based on combined mtDNA and microsatellite in Tibetan snowcock (*Tetraogallus tibetanus)*.

Groups	N(mtDNA)	H	h	π(%)	N(STR)	H_O_	H_E_	A_R_	P_A_	F_IS_
QLS	19	13	0.95	0.31	19	0.52	0.81	5.29	11	0.02
QDM	14	12	0.88	0.26	14	0.50	0.65	4.64	5	0.13
WKL	7	5	0.81	0.19	7	0.57	0.72	4.14	7	0.02
BKL	11	6	0.73	0.27	11	0.49	0.73	4.01	2	0.02
TGL	29	22	0.98	0.43	29	0.66	0.69	6.56	14	0.19

Sample sizes (N) for each group are given. For mtDNA, number of haplotypes (H); haplotype diversity (h) and nucleotide diversity as percentage () are given. For microsatellite, mean of observed heterozygosity (H_O_), expected heterozygosity (H_E_), allele richness (A_R_), number private allele (P_A_) and inbreeding coefficient (F_IS_) are indicated.

For microsatellite data, there were 7 to 21alleles per locus across all populations, and observed (H_O_) and expected heterozygosity (H_E_) ranged from 0.49 to 0.66 and 0.65 to 0.81, respectively (Table 1). F_ST_ and gene flow between all geographical groups was indicated on both mitochondrial data and microsatellite data (Table 2).

**Table 2:** Pairwise F_ST_ and gene flow among Tibetan snowcock (*Tetraogallus tibetanus)* groups based on combined mtDNA/microsatellite data, respectively.

	QLS	QDM	WKL	BKL	TGL
QLS		0.11***/0.08***	0.14***/0.11***	0.18*/0.10***	0.07***/0.04***
QDM	4.19/5.96		0.14***/0.22***	0.17/0.16***	0.07/0.10***
WKL	3.03/4.38	3.03/1.79		0.36/0.19***	0.20/0.16***
BKL	2.25/4.62	2.36/2.63	0.88/2.11		0.14/0.12***
TGL	6.26/11.72	6.25/4.28	2.02/2.59	3.21/3.60	

Above diagonal fixation indices comparing each group to the others. Below diagonal Matrix of the estimated number of migrant females/individuals per generation.

*0.01<P<0.05;

**0.001<P<0.01;

***p<0.001.

**Fig 2:**
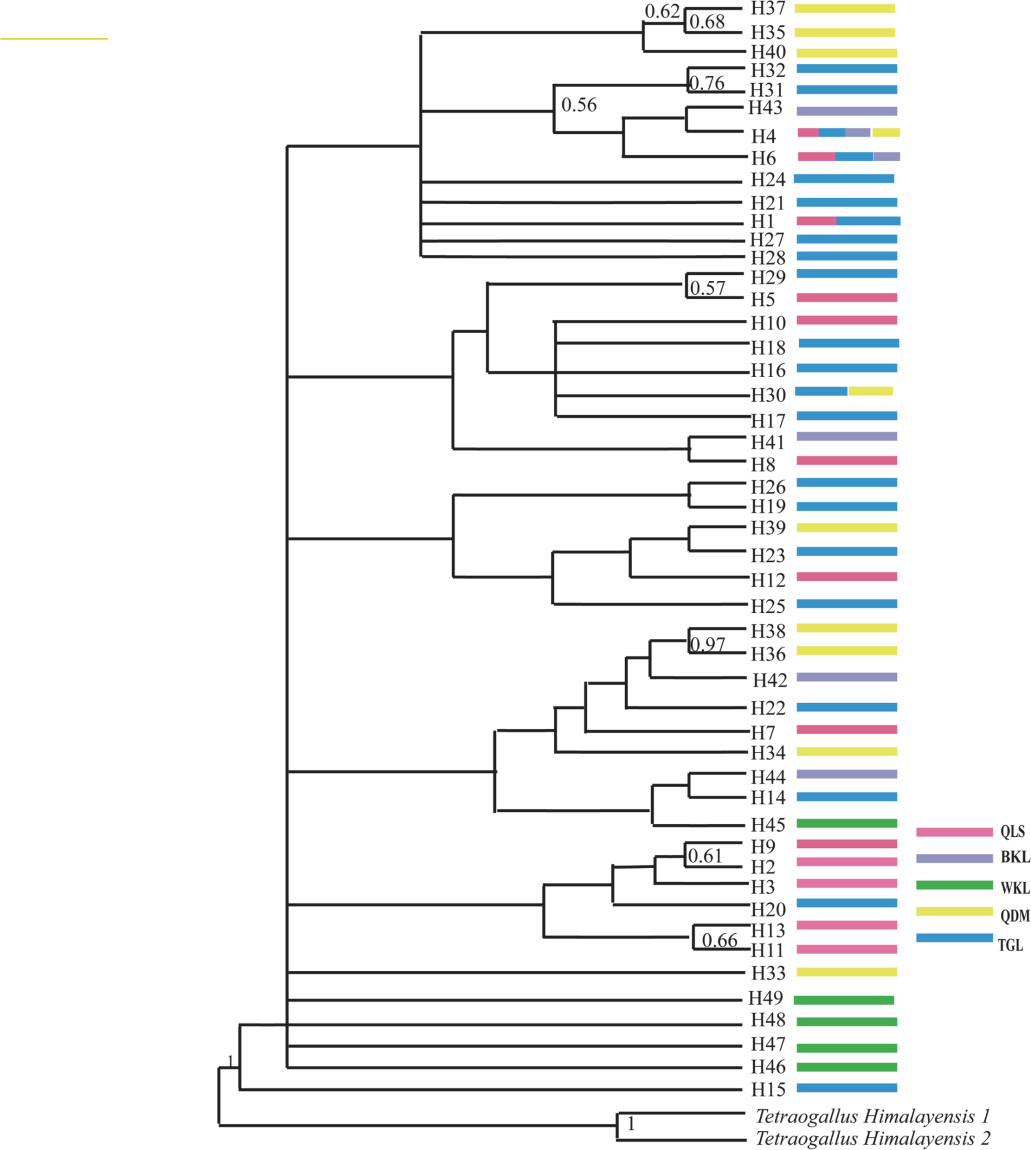
Phylogenetic relationships of all combined haplotypes identified among the Tibetan snowcock *Tetraogallus tibetanus* samples. Haplotype names are given at the terminal node of each branch in the tree. Only the Bayesian posterior probabilities above 50% are located near corresponding branches.

According to the hierarchical-likelihood ratio test under the Akaike information criterion in jMODELTEST, the GTR+I+G model were identified as the best-fitting substitution estimator, with a γ–shape correction of 0.8660. Maxium likehood phylogenetic analyses inferred with GTR+I+G model revealed limited support for phylogenetic structure (Fig 2).

**Fig 3:**
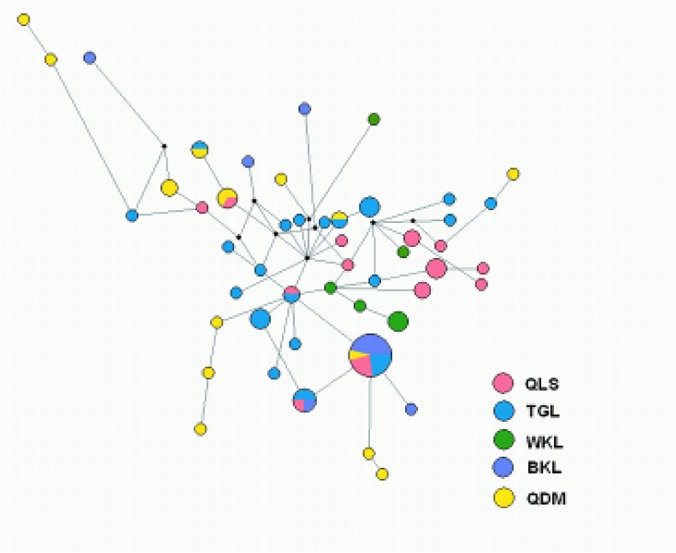
Median-joining network of combined haplotypes of Tibetan snowcock (*Tetraogallus*. *tibetanus)*. The size of the symbol is proportional to the number of individuals sharing each haplotype. The color of circles, which refer to geographical group and small black circles represent missing alleles that were not observed. A grey line between linked alleles without a number attached corresponds to one mutation.

The haplotype network generated by Median-Joining method revealed that little evidence for overt phylogeographic structure (Fig 3). The most common haplotype (H4) in network is found in four groups except WKL group (Fig 2). The presence of H4 haplotypes in four groups and not occurring in WKL group reflected WKL to be an isolated group which do not have any present connectivity with the existing other four populations. This argument could also be supported by the presence of higher number of private alleles in WKL group even with the inclusion of lesser sample size than QDM and BKL (Table 1).

The hierarchical AMOVAs revealed that most of the variations were found among individuals within sampling sites, 81.39% of all genetic variation. The components located among sampling sites were 10.49%, indicating gene flow little geographical differentiation (Table 3).

**Table 3:** Hierarchical analysis of molecular variance (AMOVA) of combined mtDNA and microsatellites among Tibetan snowcock (*T*. *tibetanus)* groups.

Variation	combined mtDNA				microsatellites			
	Variance components	Variance explained%	p-value	Fixation indices	Variance components	Variance explained%	p-value	Fixation indices
among groups	0.258	8.124	0.359	Ф_CT_ = 0.081	0.243	8.07	0.204	Ф_CT_ = 0.081
among populationswithin groups	0.334	10.49	0.000	Ф_SC_ = -0.114	0.237	7.88	0.000	Ф_SC_ = -0.086
within populations	2.593	81.39	0.0000	Ф_ST_ = 0.186	2.524	84.04	0.000	Ф_ST_ = 0.160

The AMOVA was performed using both allele frequency and molecular data simultaneously with ARLEQUIN. The p-values are the probabilities of having a more extreme variance component than the observed values by chance and are based on 1000 random permutations of the data matrix.

**Fig 4:**
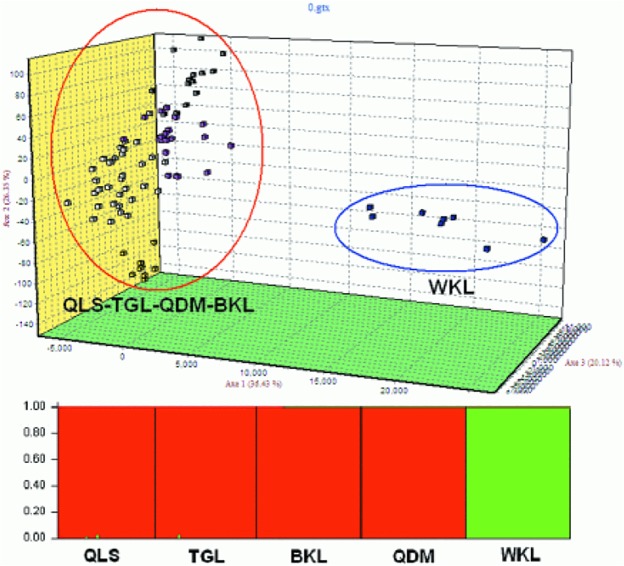
Bayesian STRUCTURE clustering results based on Tibetan snowcock (*Tetraogallus*. *tibetanus)* microsatellite genotypes of 80 individuals of Tibetan snowcock *Tibetan snowcock* from five groups. Each column along the × axis represents one *Tibetan snowcock* individual grouped by locations in the same order as in Table 1. The Y-axis represents the assignment probability of each individual into two clusters (K = 2).

The Bayesian assignment of populations to one of K = 2 clusters based on microsatellite allele frequencies gave results that were not congruent with those from the mitochondria sequences data (Fig 4). Most strikingly, the one group that contained WKL group formed a cluster. Finally, the AMOVA based on the microsatellite data indicated that 8.07% of the total genetic variation was partitioned between groups, 7.88% between populations within groups and the remaining 84.04% within populations (Table 3).

### Historical Demography

**Fig 5:**
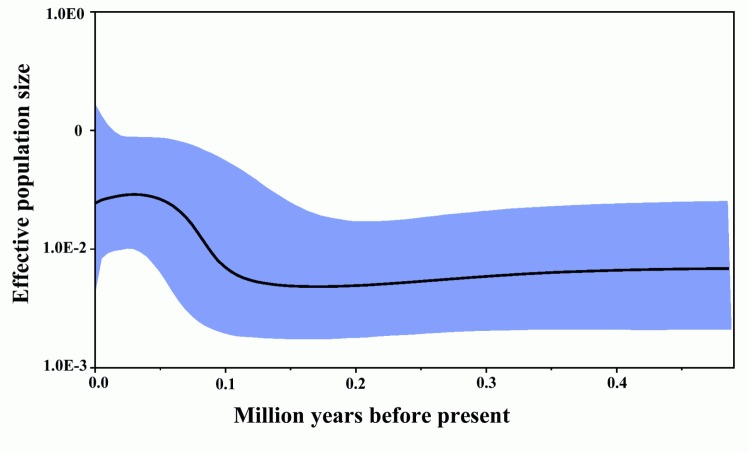
Bayesian skyline plot representing historical demographic trends in sampled Tibetan snowcock (*Tetraogallus*. *tibetanus)*. Time is reported on the x-axis as MYR (millions years ago). Estimations were based on a mutation rate of 1.6 x 10^–8^ substitutions per site per year. *Nfeτ*, the product of the effective female population size and the generation time (in years, log-transformed), is reported on the y-axis. Estimates of means are joined by a solid line; whereas dashed lines mark the 95% highest probability density limits.

Historical population trends of Tibetan snowcock inferred by the BSP seemed to fit the climate trend relatively well since the extensive glaciations period (Fig 5). Past population dynamics of this species indicated rapid population growth for the last 100 000 years. The mismatch distribution for the five groups based on mitochondrial data was bell-shaped as expected under the sudden expansion growth (S1 Fig). Fu’s test of neutrality and Tajima’s D test also produced negative values further supporting the finding of recent expansion (Table 4).

**Table 4:** Tabulated estimates of the population genetic summary statistics.

**Groups**	Θ_0_	Θ_1_	**r**	**R**	**Tajima's D**	**Fu’s Fs**
QLS	0.442	1000	0.586	0.122	-1.86**	-1.723*
QDM	2.966	1000	0.040	0.163	-1.06	-1.981*
WKL	1.986	1000	0.095	0.164	-0.30*	1.021
BKL	0.267	1000	0.013	0.243	-1.42	-0.237
TGL	0.130	1000	0.030	0.071	-1.06***	-12.66***

Θ_0_ = estimated population size before expansion, 1 = estimated population size after expansion, R = raggedness indexes), Tajima’s D values, Fu and Li’s F values.

*0.01<P<0.05;

**0.001<P<0.01;

***p<0.001

### Historical biogeography

**Fig 6:**
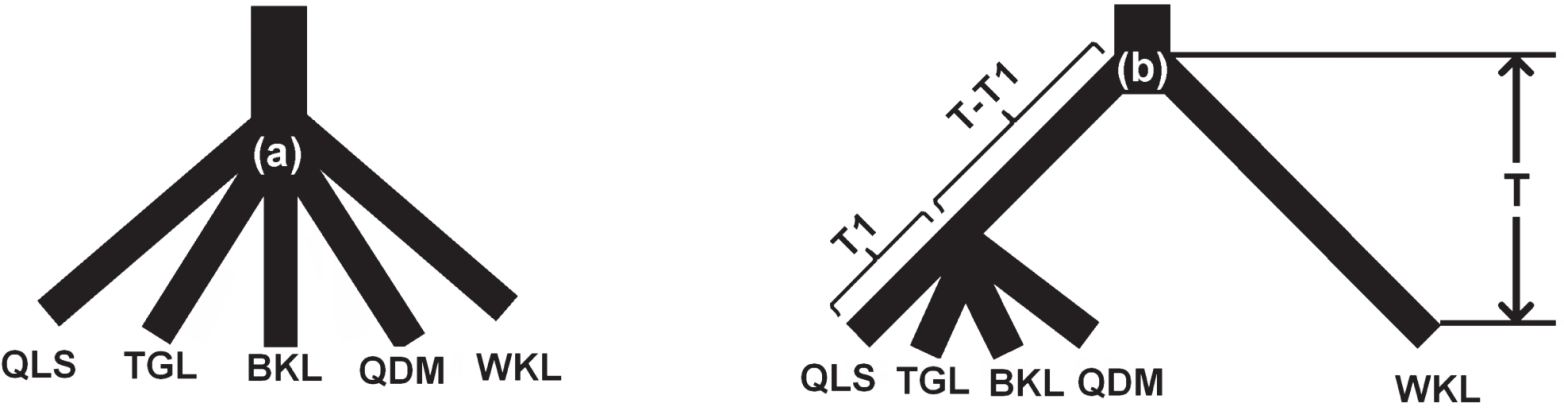
Coalescent simulations were used to test hypotheses about population structures of the Tibetan snowcock (*Tetraogallus tibetanus*). (a) The single-refugium hypothesis, in which all populations were derived from a single refugium at the end of the Last Glacial Maximum (LGM). (b) Two- or multiple-refugia hypotheses: two lineages split at the beginning of the LGM (T = 20 ka) and all current populations are derived from them with the coalescence time of T1 = 12 ka at the end of the LGM.

We conducted coalescent simulations to test two hypotheses concerning the glacial refugia of Tibetan snowcock: (1) all current populations of the species were derived from a single refugium present towards the end of the LGM (c. 12 ka) and located either at the western edge of the QTP or in the interior of the plateau (Fig 6A); (2) the species persisted during the LGM in at least two refugia, accounting for the interior plateau lineage and the plateau-edge lineage (divergence beginning 12 ka and coalescence occurring at 12 ka; Fig 6B). All gene trees were simulated within population trees with an effective population size of *Ne* = 858 481 (95% CI: 651682–1158878), which equated to a MLE estimate θ_total_ of 0.0367 with lower and upper bounds of 0.027 and 0.0496, respectively. For the observed gene tree we computed Slatin & Maddison’s S = 31.We then performed simulations under continuous migrations using the module ‘Coalescent in current tree with migration’ in Mesquite. All simulated datasets similarly rejected the first hypotheses (P < 0.01), but supported the two-refugium assumption, and the highest P-value (0.46).

## Discussion

The glacial-interglacial alteration during the Quaternary greatly influenced the present distribution pattern and phylogeographic structure of alpine species of QTP [18]. The Pleistocene glaciations cycles are considered to have been critical in shaping the distributions and genetic attributes of most species [5,9]. During the glaciation extremes, species were forced into isolated refugia, thereby enhancing intraspecific differentiation. This is a commonly observed phenomenon for many terrestrial species that were unable to withstand the cold temperature conditions. Many previous studies conducted in QTP demonstrated that species retreated to refugia in the peripheral area during glaciations and two main glacial refugia were identified in QTP, namely along the eastern and south-eastern edges [2,3,5,18]. However, for species those have become adapted to high-elevation environments, such as Tibetan snowcock, the glaciations may have had the opposite effect of enhancing the interactions among populations. Our mtDNA sequences data indicate Tibetan snowcock populations inhabiting montane habitats with current species range, suggesting that Tibetan snowcock survived in the central of QTP similar to other species such as the cold-tolerant the plateau zokor *Eospalax baileyi* [7], an alpine shrub *Potentilla fruticosa* [41] and an alpine plant *Aconitum gymnandrum* [42].

The phylogeographic analysis revealed shallow divergence among haplotypes and no clear substructure and the starlike topology of the median-joining network both indicate sudden population expansion from several refugia, probably located in the TGL, QDM (Fig 4). The whole samples and five groups displayed unimodal distributions and Fu’s *Fs* and Tajima’s D statistic were negative expected under rapid population growth at about10,000 year ag (Table 4 and S1 Fig). And coincide well with the Bayeasian sky plot (Fig 5). Populations from refugia often show high genetic diversity due to refugia persistence and accumulation of variation [2,43]. In agreement with this prediction, TGL and QDM from Tonggula Mountains in the central of QTP displayed slightly higher nucleotide richness in mitochondria DNA and allele richness in microsatellite (Table 1). Furthermore, additional haplogroups, statistically supported, emerged on the Bayesian analyses and the Median-joining network also showed long branches. These long branches notably lead to haplogroups located in the central part of the Qinghai-Tibetan Plateau, suggesting that at least other refugia may have existed QDM. Populations from recently glaciated regions should display relatively low genetic diversity when compared with stable populations persisting in refugia, and colonizing populations should bear signatures of population expansion following the dispersal route. The persistence of Tibetan snowcock populations at such during the Last Glacial Maximum is further supported by the fact that all the investigated groups occur at high altitudes (>3700 m). The existence such glacial refugium in central of QTP is also proved by the presence of cold-tolerant alpine species *A*. *gymnandrum*, *E*. *baileyi* and *P*. *fruticosa* [7,41,42].

Moreover, landscape features also can deeply impact the partitioning of the genetic diversity and gene flow between populations especially across increasingly fragmented habitats [44,45]. However, previously identified barriers across western QTP appear to have had little impact on Tibetan snowcock. Hence, it is not unexpected that glacier-covered mountain ridges and predominately rocky habitats do not strongly impact gene flow as suggested by both mitochondrial and nuclear DNA markers microsatellite. Many montane-adapted species experienced range expansions during cooler glacial cycles allowing for increased population connectivity and gene flow [46]. Generally, populations experiencing long-term isolation in separated refugia during climatic oscillations tend to show a sign of strong genetic divergence among refugia [47]. In contrast, gene flow may erase any evidence of divergence due to allopatry, and genetic divergence among populations might be low if cyclical fluctuations have allowed recurrent population connectivity. The high elevation meadow and steppe habitats were probably less fragmented during the glacial advance than in the present and several alpine species were recorded at high altitude in the QTP [48]. Given that typical habitats of Tibetan snowcock are found above the snow line, isolated populations within the species’ range might be connected during cold periods. Hence, different from most of other avian taxa, Tibetan snowcock probably increased its distribution range during the glaciations. Noteworthy, this pattern coincides with that of Montagu’s Harrier (Circus pygargus) whose connection among groups might have been maximal during the long glacial periods, being interrupted only during the shorter interglacial phases [43]. Interestingly, our results suggest that the interglacial period might have been too short for genetic divergence among refugia to arise. Tibetan snowcock is thus currently in its contraction phase, and it has been suggested that current-day and future warmer conditions may represent their refugia phases like other cold-tolerant species [49]. These events were likely associated with vegetation shifts caused by climate and human-induced changes during the Holocene, which can be proved by its expansion time is similar to *P*. *fruticosa* [41] and an alpine herb *Pedicularis longifolra* [50] on the Qinghai-Tibetan Plateau.

Geographic complexity and environmental heterogeneity are likely to have shaped the genetic structure among Tibetan snowcock populations. Such a landscape effect is evident in the western and central of QTP. Although Tibetan snowcock has a rather restricted geographic distribution, mitochondrial data support the intermixing lineage with poor support, while microsatellites indicate a clear subdivision into a western and a central lineages. The discordance between mitochondria and microsatellite data is most likely explained by the different mutation rate of these markers, which reflects different processes acting at different times in the evolution of this species. We speculate this maybe is the evidence of ongoing divergent process. This pattern of population divergence between western Kunlun Mountains and central of QTP is consistent with the other dispersal-limited bird the twite (*Carduelis flavirostris*)[51]. Although our data disclosed some interesting results, many factors probably affect expansion timing estimates and patterns including mutation rates, generation time and genetic loci. Increased sampling may clarify our interpretations.

The Qinghai-Tibetan Plateau is well known as the highest plateau in the world and one of world biodiversity hotspot. As occurring on the plateau and adjacent regions, Tibetan snowcock showed lack of evidently phylogeographic structure on mitochondria data and historical population expansion, suggesting instead historically wide gene flow and a relatively stable demography, as found in other plateau species or populations [52]. This research provided additional evidence that specialization to cold habitat promotes gene flow and reduces population genetic differentiation in QTP. Hopefully, we wish to improve this work by exploring patterns of population differentiation in other avian taxa that are specialized to cold environment and high altitudes.

## Supporting Information

S1 Fig Mismatch distribution for mitochondria DNA. The solid line represents the expected distribution; the dashed line represents the observed distribution. Mismatch distribution for the following groups are shown: QLS, QDM, WKL, BKL and TGL.
(TIF)

S1 Table Information of Tibetan snowcock (*Tetragallus tibetanus*) samples used in this study. All vouchers are deposited in the Lanzhou University. Location names correspond to those on the map in Fig 1.
(DOC)

S2 Table Sequences of primers used in PCR amplification a sequencing of Tibetan snowcock (*Tetraogallus*. *tibetanus)*. (DOC)
