# Correction: Refugia Persistence of Qinghai-Tibetan Plateau by the Cold-Tolerant Bird *Tetraogallus tibetanus* (Galliformes: Phasianidae)

**DOI:** 10.1371/journal.pone.0130098

**Published:** 2015-06-03

**Authors:** Bei An, Lixun Zhang, Naifa Liu, Ying Wang

In the Funding section, the grant numbers from the funder National Natural Science Foundation of China are listed incorrectly. The correct grant numbers are: 31372195 and 31101625.
