# Supplementary figures and images for: Refugia Persistence of Qinghai-Tibetan Plateau by the Cold-Tolerant Bird Tetraogallus tibetanus (Galliformes: Phasianidae)
Source: PLoS One. 2015 Mar 30;10(3):e0121118. doi: 10.1371/journal.pone.0121118 (PMC4378977; doi:10.1371/journal.pone.0121118)

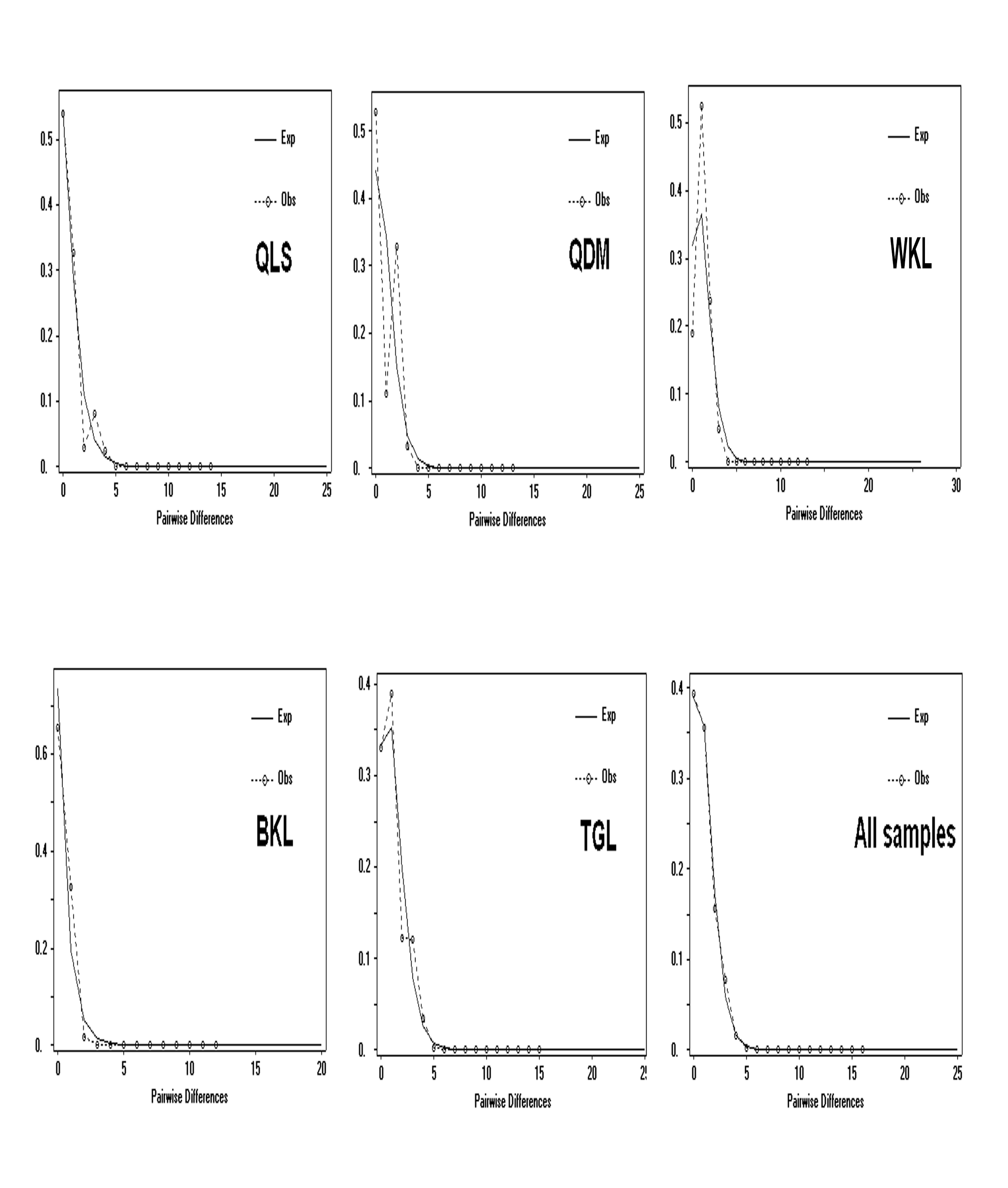

Supplement: S1 Fig — The solid line represents the expected distribution; the dashed line represents the observed distribution. Mismatch distribution for the following groups are shown: QLS, QDM, WKL, BKL and TGL. (TIF) [file pone.0121118.s001.tif]
