# Supplementary material for: Refugia Persistence of Qinghai-Tibetan Plateau by the Cold-Tolerant Bird Tetraogallus tibetanus (Galliformes: Phasianidae)
Source: PLoS One. 2015 Mar 30;10(3):e0121118. doi: 10.1371/journal.pone.0121118 (PMC4378977; doi:10.1371/journal.pone.0121118)
Supplement: S1 Table — All vouchers are deposited in the Lanzhou University. Location names correspond to those on the map in Fig 1. (DOC) [file pone.0121118.s002.doc]

**Table S1**. Information of Tibetan snowcock (*Tetragallus tibetanus*) samples used in this study. All vouchers are deposited in the Lanzhou University. Location names correspond to those on the map in Fig.1.

| Group | Population/Location | Altitude(m) | Latitude | Longtitude | Sample size | Type of sample |
| --- | --- | --- | --- | --- | --- | --- |
|  |  |  |  |  |  |  |
| QLS (n = 19) | Datong, DT | 3740 | 36°54'N | 101°30'E | 6 | Liver |
|  | Sunan, SN | 4160 | 38°50'N | 99°26'E | 6 | Muscle |
|  | Tianzhu ,TZ | 3860 | 37°11'N | 103°08'E | 7 | Liver |
| WKL (n = 7) | Atushi, ATS | 3960 | 40°18'N | 75°37'E | 4 | Muscle |
|  | Wuqia, WQ | 4205 | 39°53'N | 75°12'E | 2 | Muscle |
|  | Yecheng, YC | 4180 | 37°14'N | 76°33'E | 1 | Muscle |
| QDM (n =14) | Haixi, HX | 4120 | 37°28'N | 97°27'E | 6 | Liver |
|  | Delingha, DLH | 3900 | 37°14'N | 98°10'E | 8 | Liver |
| TGL (n = 29) | Zhiduo, ZD | 4500 | 33°54'N | 95°38'E | 6 | Liver |
|  | Biru, BR | 4300 | 31°24'N | 93°39'E | 2 | Liver |
|  | Baqin, BQ | 4530 | 31°58'N | 94°04'E | 5 | Muscle |
|  | Anduo, AD | 4840 | 32°03'N | 91°46'E | 14 | Liver |
|  | Suoxian, SX | 4665 | 31°50'N | 93°36'E | 2 | Liver |
| BKL (n = 11) | Qumalai, QML | 4850 | 34°08'N | 95°56'E | 11 | Liver |
| Total | 14 |  |  |  | 80 |  |
