# Supplementary material for: Refugia Persistence of Qinghai-Tibetan Plateau by the Cold-Tolerant Bird Tetraogallus tibetanus (Galliformes: Phasianidae)
Source: PLoS One. 2015 Mar 30;10(3):e0121118. doi: 10.1371/journal.pone.0121118 (PMC4378977; doi:10.1371/journal.pone.0121118)
Supplement: S2 Table — (DOC) [file pone.0121118.s003.doc]

**Table S2.** Sequences of primers used in PCR amplification a sequencing of Tibetan snowcock (*Tetraogallus .tibetanus)*.

| Primer names | Expected size(bp) | Forward Primer (5 to 3’) | Reverse Primer (5 to 3’) | Annealing temperature(℃) |
| --- | --- | --- | --- | --- |
| CR | 854 | GGGAAACTATGCATAATCGTGC | AGTGGAGTTTCTCTAATAATGTAGG | 53 |
| *Cyt* b | 705 | CTACCATGAGGACAAATATC | TCTTGGCATCTTCAGTGCCATGC | 57.5 |
|  |  |  |  |  |
